# Effect of Chemotherapy on Overall Survival in Contemporary Metastatic Prostate Cancer Patients

**DOI:** 10.3389/fonc.2021.778858

**Published:** 2021-11-23

**Authors:** Benedikt Hoeh, Christoph Würnschimmel, Rocco S. Flammia, Benedikt Horlemann, Gabriele Sorce, Francesco Chierigo, Zhe Tian, Fred Saad, Markus Graefen, Michele Gallucci, Alberto Briganti, Carlo Terrone, Shahrokh F. Shariat, Derya Tilki, Luis A. Kluth, Philipp Mandel, Felix K. H. Chun, Pierre I. Karakiewicz

**Affiliations:** ^1^ Department of Urology, University Hospital Frankfurt, Goethe University Frankfurt am Main, Frankfurt am Main, Germany; ^2^ Cancer Prognostics and Health Outcomes Unit, Division of Urology, University of Montréal Health Center, Montréal, QC, Canada; ^3^ Martini-Klinik Prostate Cancer Center, University Hospital Hamburg-Eppendorf, Hamburg, Germany; ^4^ Department of Maternal-Child and Urological Sciences, Sapienza Rome University, Policlinico Umberto I Hospital, Rome, Italy; ^5^ Division of Experimental Oncology/Unit of Urology, Urological Research Institute, San Raffaele Scientific Institute, Milan, Italy; ^6^ Department of Surgical and Diagnostic Integrated Sciences (DISC), University of Genova, Genova, Italy; ^7^ Department of Urology, Comprehensive Cancer Center, Medical University of Vienna, Vienna, Austria; ^8^ Department of Urology, Weill Cornell Medical College, New York, NY, United States; ^9^ Department of Urology, University of Texas Southwestern, Dallas, TX, United States; ^10^ Department of Urology, Second Faculty of Medicine, Charles University, Prague, Czechia; ^11^ Institute for Urology and Reproductive Health, Sechenov First Moscow State Medical University, Moscow, Russia; ^12^ Division of Urology, Department of Special Surgery, Jordan University Hospital, The University of Jordan, Amman, Jordan; ^13^ Department of Urology, University Hospital Hamburg-Eppendorf, Hamburg, Germany

**Keywords:** chemotherapy, overall survival, metastatic prostate cancer, SEER, contemporary

## Abstract

**Introduction:**

Randomized clinical trials demonstrated improved overall survival in chemotherapy exposed metastatic prostate cancer patients. However, real-world data validating this effect with large scale epidemiological data sets are scarce and might not agree with trials. We tested this hypothesis.

**Materials and Methods:**

We identified *de novo* metastatic prostate cancer patients within the Surveillance, Epidemiology, and End Results (SEER) database (2014-2015). Kaplan-Meier plots and Cox regression models tested for overall survival differences between chemotherapy-exposed patients *vs* chemotherapy-naïve patients. All analyses were repeated in propensity-score matched cohorts. Additionally, landmark analyses were applied to account for potential immortal time bias.

**Results:**

Overall, 4295 *de novo* metastatic prostate cancer patients were identified. Of those, 905 (21.1%) patients received chemotherapy *vs* 3390 (78.9%) did not. Median overall survival was not reached at 30 months follow-up. Chemotherapy-exposed patients exhibited significantly better overall survival (61.6 *vs* 54.3%, multivariable HR:0.82, CI: 0.72-0.96, p=0.01) at 30 months compared to their chemotherapy-naïve counterparts. These findings were confirmed in propensity score matched analyses (multivariable HR: 0.77, CI:0.66-0.90, p<0.001). Results remained unchanged after landmark analyses were applied in propensity score matched population.

**Conclusions:**

In this contemporary real-world population-based cohort, chemotherapy for metastatic prostate cancer patients was associated with better overall survival. However, the magnitude of overall survival benefit was not comparable to phase 3 trials.

## Introduction

Systemic treatments for metastatic prostate cancer have grown exponentially over the last two decades and exhibited significant survival benefits in randomized phase 3 trials (1–8). However, trial findings may be difficult to replicate in real-world conditions. Indeed, only one report demonstrated a modest benefit in overall survival after chemotherapy in contemporary, *de novo* metastatic prostate cancer patients (Weiner et al., National Cancer Database 2014-2015) (9). We addressed the same endpoint within the same study period. Within a different, large-scale database (SEER), we focused on the most contemporary patients (2014-2015) diagnosed with *de novo* metastatic prostate cancer. We hypothesized that chemotherapy use may result in a survival benefit for *de novo* metastatic prostate cancer patients (9). Unlike Weiner et al., we relied on propensity score matching to maximally reduce potential differences between chemotherapy-exposed and chemotherapy-naïve patients.

## Material and Methods

### Study Population

The current SEER database samples 34.6% of the United States population and approximates it in demographic composition and cancer incidence (10). Within the SEER database (2014-2015), we identified patients ≥18 years old with *de novo* metastatic, histologically confirmed adenocarcinoma of the prostate, diagnosed at biopsy (International Classification of Disease for Oncology [ICD‐O‐3] code 8140 site code C61.9) between 2014 and 2015. Patients with unknown M-stage, cases identified at autopsy or through death certificates, with unknown histology or non-primary prostate cancers were excluded. These selection criteria resulted in a cohort of 4295 *de novo* metastatic prostate cancer patients. This subgroup represented the study population.

### Statistical Analyses

The statistical analyses consisted of four steps. First, we addressed overall survival prior to propensity score matching. We relied on Kaplan-Meier plots and Cox regression models to test for overall mortality differences according to chemotherapy exposure. Covariates consisted of age at diagnosis, PSA groups (<20, 20-90, >90 in ng/ml), Gleason Group Grade (GGG) at biopsy (≤III, IV/V, unknown), clinical T-stage (≤cT2, cT3/4, cTx), clinical N-stage (cN0, cN1, cNx), clinical M-stage (cM1a/b, cM1c, cM1x) and type of local treatment (no local treatment, local treatment, unknown).

Second, we relied on propensity score matching to address potential differences between chemotherapy-exposed *vs* chemotherapy-naïve patients using the ‘nearest neighbor’ and a caliper of 0.05. Matching variables consisted of age (per year interval), PSA (<20, 20-90, >90 in ng/ml), GGG (I, II, III, IV, V, unknown), T-stage (cT1, cT2, cT3, cT4, cTx), N-stage (cN0, cN1, cNx), M-stage (cM1a, cM1b, cM1c, cM1unspecific) socioeconomic status (1^st^, 2^nd^/3^rd^/4^th^ quartile) and type of local treatment (RP, RT, RP+RT, none). Each chemotherapy exposed patient was matched to two chemotherapy naïve patient. Third, we relied on the propensity score matched cohorts of chemotherapy-exposed and chemotherapy-naïve patients and refitted Kaplan-Meier plots, as well as multivariable Cox regression models. The same covariates were used as above. Finally, survival analyses were repeated in propensity score matched cohorts after landmark analyses (3 months) was applied to account for confounding effects due to potential immortal time bias.

All tests were two sided with a level of significance set at p<0.05 and R software environment for statistical computing and graphics (version 3.4.3) was used for all analyses (11).

## Results

### Descriptive Characteristics of Study Population

Between 2014 and 2015 we identified 4295 *de novo* metastatic prostate cancer patients. Of those, 905 patients (21.1%) received chemotherapy. Chemotherapy-exposed patients differed from their chemotherapy naïve counterparts with respect to age (64 *vs* 70 years, p<0.001), higher proportions of PSA >90 ng/ml (57.3 *vs* 51.8%, p=0.01), higher proportions of GGG V (52.3 *vs* 43.6%, p=0.01), higher proportions of cN1-stages (44.5 *vs* 31.6%, p<0.001) and higher proportions of cM1c-stages (19.8 *vs* 14.6%, p<0.001). No significant differences were recorded for type of local treatment.

### Survival Analyses Without Propensity Score Matching

**Figure 1:**
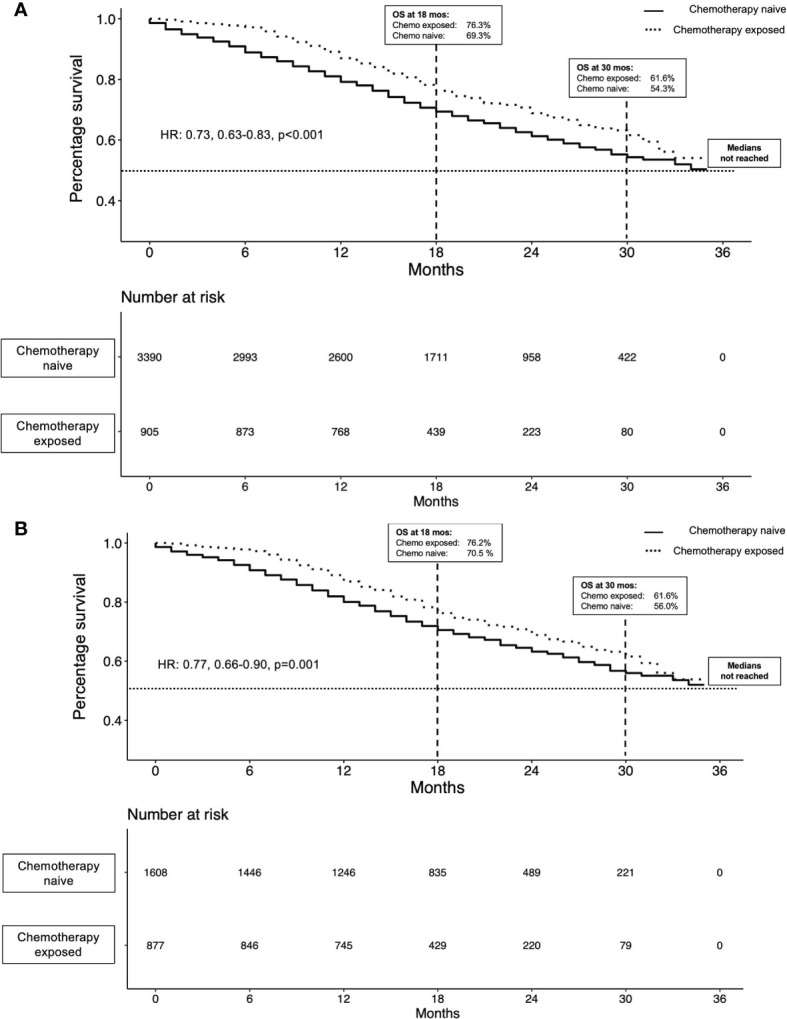
Kaplan-Meier plots illustrating overall survival in metastatic prostate cancer (mPCa) patients (n=2495) prior to propensity score matching **(A)** and in 2490 mPCa patients after propensity score matching **(B)**, stratified by chemotherapy status.

Based on the overall cohort, that included 905 chemotherapy-exposed *vs* 3390 chemotherapy-naïve patients, overall survival rates at 18 and 30 months were 76.3 *vs* 69.3% and 61.6 *vs* 54.3%, favoring chemotherapy-exposed patients (**Figure 1A**). In multivariable Cox regression models, chemotherapy exposed patients exhibited lower overall mortality (HR:0.82, CI: 0.72-0.96, p=0.01) compared to chemotherapy naïve patients (**Table 2**).

### Propensity Score Matching

Propensity score matching focused on the overall study cohort, of who 905 chemotherapy-exposed *vs* 3390 chemotherapy-naïve patients. Of 905 chemotherapy-exposed patients, 879 could be matched with up two chemotherapy-naïve patients, which resulted in two subgroups, respectively with 879 chemotherapy-exposed *vs* 1611 chemotherapy-naïve patients. No statistically significant differences in age at diagnosis, PSA groups, GGG, cT-stage, cN-stage, cM-stage, SES and approach of local treatment remained between these two cohorts (all p≥0.1; **Table 1**).

**Table 1:** Descriptive characteristics of *de novo* metastatic prostate cancer patients between 2014 and 2015, stratified by chemotherapy exposure.

	Unmatched data				Propensity score matched data			
	Overall, (n = 4295)	Chemotherapy naïve, (n = 3390)	Chemotherapy exposed, (n = 905)	p-Value	Overall (n = 2490)	Chemotherapy naïve, (n = 1611)	Chemotherapy exposed, (n = 879)	p-Value
**Age in yrs** median (IQR)	69 (61-77)	70 (63-79)	64 (58-70)	<0.001	64 (59-71)	64 (59-71)	64 (58-71)	0.1
**PSA-groups in ng/ml** n (%)				0.01				0.5
low (<20)	899 (20.9)	732 (21.6)	167 (18.5)		479 (19.3)	315 (19.6)	164 (18.7)	
intermediate (21-90)	1120 (26.1)	901 (26.6)	219 (24.2)		628 (25.3)	415 (25.8)	213 (24.3)	
high (>90)	2276 (53)	1757 (51.8)	519 (57.3)		1378 (55.5)	878 (54.6)	500 (57)	
**GGG Biopsy** n (%)				<0.001				1.0
I	59 (1.4)	51 (1.5)	8 (0.9)		26 (1)	18 (1.1)	8 (0.9)	
II	162 (3.8)	146 (4.3)	16 (1.8)		43 (1.7)	27 (1.7)	16 (1.8)	
III	312 (7.3)	261 (7.7)	51 (5.6)		151 (6.1)	100 (6.2)	51 (5.8)	
IV	799 (18.6)	646 (19.1)	153 (16.9)		436 (17.5)	284 (17.7)	152 (17.3)	
V	1951 (45.4)	1478 (43.6)	473 (52.3)		1284 (51.7)	831 (51.7)	453 (51.7)	
Unknown	1012 (23.6)	808 (23.8)	204 (22.5)		545 (21.9)	348 (21.6)	197 (22.5)	
**cT-stage** n (%)				0.3				0.9
cT1	1212 (28.2)	960 (28.3)	252 (27.8)		697 (28)	455 (28.3)	242 (27.6)	
cT2	1212 (28.2)	952 (28.1)	260 (28.7)		720 (29)	466 (29)	254 (29)	
cT3	466 (10.8)	366 (10.8)	100 (11)		289 (11.6)	190 (11.8)	99 (11.3)	
cT4	561 (13.1)	428 (12.6)	133 (14.7)		337 (13.6)	210 (13.1)	127 (14.5)	
cTx	844 (19.7)	684 (20.2)	160 (17.7)		442 (17.8)	287 (17.8)	155 (17.7)	
**cN-stage** n (%)				<0.001				0.6
cN0	2208 (51.4)	1805 (53.2)	403 (44.5)		1142 (46)	743 (46.2)	399 (45.5)	
cN1	1473 (34.3)	1070 (31.6)	403 (44.5)		1047 (42.1)	667 (41.5)	380 (43.3)	
cNX	614 (14.3)	515 (15.2)	99 (10.9)		296 (11.9)	198 (12.3)	98 (11.2)	
**M-stage** n (%)				<0.001				0.9
M1a	324 (7.5)	273 (8.1)	51 (5.6)		154 (6.2)	148 (6)	97 (6)	
M1b	3200 (74.5)	2539 (74.9)	661 (73)		1847 (74.2)	1849 (74.4)	1201 (74.7)	
M1c	674 (15.7)	495 (14.6)	179 (19.8)		451 (18.1)	445 (17.9)	281 (17.5)	
M1x	97 (2.3)	83 (2.4)	14 (1.5)		51 (1.7)	148 (6)	97 (6)	
**Socioeconomic status** n (%)				0.04				0.6
1^st^ quartile	1082 (25.2)	830 (24.5)	252 (27.8)		668 (26.9)	426 (26.5)	242 (27.6)	
2^nd^-4^th^ quartile	3213 (74.8)	2560 (75.5)	653 (72.2)		1817 (73.1)	1182 (73.5)	635 (72.4)	
**Local treatment** n (%)				0.2				1.0
None	3079 (71.7)	2419 (71.4)	660 (72.9)		1797 (72.3)	1157 (72)	640 (73)	
RP	99 (2.3)	88 (2.6)	11 (1.2)		36 (1.4)	25 (1.6)	11 (1.3)	
RT	843 (19.6)	664 (19.6)	179 (19.8)		489 (19.7)	318 (19.8)	171 (19.5)	
RP+RT	182 (4.2)	144 (4.2)	38 (4.2)		111 (4.5)	73 (4.5)	38 (4.3)	
Unknown	92 (2.1)	75 (2.2)	17 (1.9)		52 (2.1)	35 (2.2)	17 (1.9)	

All values are median (IQR) or frequencies (%).

RP, Radical prostatectomy; RT, Radiotherapy.

### Survival Analyses After Propensity Score Matching

Based on the propensity matched cohorts of 879 chemotherapy-exposed *vs* 1611 chemotherapy-naïve patients, overall survival rates at 18 and 30 months were 76.3 *vs* 70.5% and 61.6 *vs* 56.0%, favoring chemotherapy-exposed patients (**Figure 1B**).

In multivariable Cox regression models, chemotherapy exposed patients exhibited lower overall mortality (HR:0.77, CI: 0.66-0.90, p<0.001) compared to chemotherapy naïve patients (**Table 2**). The effect of better survival in chemotherapy-exposed remained unchanged after landmark analyses was applied in the propensity score matched cohort (multivariable HR: 0.85; CI: 0.72-0.99; p=0.04).

**Table 2:** Multivariable Cox regression models predicting overall mortality in *de novo* metastatic prostate cancer patients according to chemotherapy status prior to and after propensity score matching.

	Variable of interest	Univariable			Multivariable		
		Hazard Ratio	95%-CI	p-value	Hazard Ratio	95%-CI	p-value
**Unmatched data**	chemotherapy-exposed *vs*. naïve	0.73	0.63-0.83	<0.001	0.82	0.72-0.96	0.01
**After propensity score matching**	chemotherapy-exposed *vs*. naïve	0.77	0.66-0.90	0.001	0.77	0.66-0.90	<0.001

Cox regression models were adjusted for age, PSA, Gleason Group Grade, cT-stage, cN-stage, cM-stage and local treatment.

## Discussion

We hypothesized that, in line with trial-derived findings and smaller population-based studies, chemotherapy exposed *de novo* metastatic prostate cancer patients exhibit better survival rates compared to their chemotherapy naïve counterparts. We tested this hypothesis within a large population-based cohort *de novo* metastatic prostate cancer patients diagnosed between 2014 and 2015.

First, we observed significantly worse cancer characteristics in chemotherapy-exposed patients compared to their chemotherapy naïve counterparts. Specifically, they exhibited higher proportions of high PSA, higher proportions of GGG V, higher proportions of cN1-stage and higher proportions of cM1c-stage. It is of note that despite an obvious prostate cancer phenotype disadvantage in chemotherapy-exposed prostate cancer patients, their overall survival was better, as will be outlined below. These observations are similar to NCDB patient characteristics. In consequence, it may be postulated that both databases (NCDB and SEER) indicate that chemotherapy is offered to patients with more aggressive prostate cancer phenotype than average (9). The same observations regarding prostate cancer characteristics were made in smaller scale, retrospective studies (9, 12).

Second, within the current study cohort the rate of chemotherapy was 21.1% (n=905) *de novo* metastatic prostate cancer patients. This proportion is disappointingly low, however it is very comparable to NCDB, where chemotherapy was also given to a minority of patients (27.6%). Similarly low rates were recorded in other, smaller scale population-based studies (12, 13). These observations indicate a relatively low confidence level in systemic therapy. Additionally, risk of chemotherapy-related adverse events, which vary in regard to the dose and type of chemotherapeutical agent administered, may result in tendencies towards more restrictive chemotherapy administration policies. Even though that recent studies have recorded an increase of chemotherapy rates in more contemporary years (14, 15), efforts are further required to encourage referrals from within the urological community for systemic therapy, when metastatic prostate cancer is diagnosed (1).

Third, we recorded more favorable survival in chemotherapy-exposed *vs* chemotherapy-naïve patients (76.3 *vs* 69.3% and 61.6 *vs* 54.3% at 18 and 30 months). These rates resulted in a highly protective multivariable hazard ratio of 0.82 (CI:0.72-0.96, p=0.01). Finally, even after detailed propensity score matching for differences in patient and prostate cancer characteristics, a protective hazard ratio of 0.77 (CI:0.66-0.90, p<0.001) was recorded. Additionally, to propensity score matching, we furthermore repeated the survival analyses after landmark analyses was applied to maximally reduce potential biases that might have occurred due to immortal time biases. Irrespectively of these two strict methodological approaches to maximally reduce any biases which may arise from differences between chemotherapy exposed *vs* naïve *de novo* mPCa patients, survival trends remained in its quantity and quality unchanged.

These observations are highly consistent with NCDB-derived findings on the same topic (Weiner et al.) (9). Conversely, to the best of our knowledge, no other reports identified a survival benefit in contemporary, metastatic prostate cancer patients exposed to chemotherapy compared to their chemotherapy naïve counterparts. In consequence, it may be postulated that the survival benefit only became apparent in the most contemporary population-based metastatic prostate cancer patients, in both the SEER and the NCDB. To the best of our knowledge, prior to Weiner et al. and to the current study, a formal comparison between chemotherapy-exposed *vs* chemotherapy-naïve patients was not reported. Instead, previous population-based analyses examined survival trends regardless of chemotherapy exposure status. These trends exhibited only marginal improvement over time (14). For example, Cattrini et al. reported only a modest improvement of median overall survival (30 *vs* 26 months) in contemporary (2011-2014) metastatic prostate cancer patients in comparison to historical (2000-2003) metastatic prostate cancer patients exposed to chemotherapy. Since Cattrini et al. did not furthermore account for any treatment approach and primarily focused on the cohort of metastatic prostate cancer patients from an epidemiological aspect, results cannot directly be compared to the current study (16). In consequence, the current study, as well as the study by Weiner et al., cannot be directly compared to previous population-based studies with different designs and endpoints. Similarly, our findings cannot be directly compared to phase 3 trials, that focused on specific molecules and treatment regimens in randomized designs. In the current and Weiner et al. studies chemotherapy-exposed patients may have received one or multiple chemotherapy lines. Unfortunately, their specific time and duration of exposure is unknown in the current study, as well as in the Weiner et al. study. Consequently, some chemotherapy-exposed patients may have received a single line of chemotherapy with no overall survival benefit. Conversely, others may have received multiple lines with an important overall survival benefit. It is of note that combination therapies, including chemotherapeutic agents, are likely to play an important role in the near future. Recently, results derived from the PEACE-1 trial demonstrated for example that addition of abiraterone to androgen deprivation therapy (ADT) and docetaxel significantly improved radiographic progression-free survival in *de novo* metastatic castration sensitive prostate cancer patients (17). Last, but not least, the current study differed from Weiner et al. in its design. We relied on propensity score matching to maximally attenuate differences between chemotherapy-exposed and chemotherapy-naïve metastatic prostate cancer patients. Despite propensity score matching use in the current study, the previously recorded overall survival benefit observed in chemotherapy-exposed patients remained in the current analyses. Similarly, its magnitude remained virtually unchanged. It is noteworthy, that the magnitude of the benefit in the current study, as well as in the study of Weiner et al., cannot be directly compared to the magnitude of survival benefit recorded in phase 3 trials for specific systemic approaches for metastatic prostate cancer (18, 19). It is of note, that the magnitude of the survival benefit in most of phase 3 studies addressing overall survival in metastatic prostate cancer was greater than the magnitude recorded in our study, as well as that recorded in the study of Weiner et al. and other small scale institutional studies (20–22).

Regardless of the very important beneficial survival rates in chemotherapy exposed metastatic prostate cancer patients in respect to chemotherapy naïve patients, several limitations need to be acknowledged. First, the rate of chemotherapy exposure is low in the current study. It is nonetheless very similar to the rate observed in the study of Weiner et al. Moreover, the nature of administered chemotherapy is unknown with respect to the number of lines, their duration, as well as their individual efficacy. Furthermore, treatment approaches such as palliative care or observational approaches, are not available in the SEER database. Therefore, potential biases which may have occurred due to different supportive care measurements cannot be ruled out and should be taken into account when data is interpreted. Similar to Weiner et al., we could not adjust or circumvent these limitations.

Second, the retrospective nature of the study introduces a number of selection biases, that distinguish chemotherapy exposed patients from others. As reported, chemotherapy-exposed patients tended to harbor more aggressive prostate cancer phenotypes. The same limitation applied to the study cohort focusing on NCDB. In the Weiner et al. study, these differences were addressed in multivariable analyses. Conversely, in the current study, multivariable analyses were complemented by propensity score matching to more completely and strictly address these differences.

Third, certain additional unmeasured variables could not be addressed. These variables, including performance status and comorbidities, were unavailable in the current study. Some of these variables, including comorbidities, were available in the Weiner et al. study (9). Despite their availability, overall survival rates virtually perfectly agreed with rates recorded in the current study. In consequence, lack of comorbidities does not appear to represent a rate limiting factor. Fourth, strict stratification according to low- and high-volume tumor burden, as performed in previously reported phase-3 trials is limited by the nature of SEER data collection (18). Finally, a number of established predictors of survival (Lactate dehydrogenase, hemoglobin) for metastatic prostate cancer patients were unavailable in both the current and NCDB analyses (23).

## Conclusions

In the largest contemporary, North-American population-based study, chemotherapy exposure for metastatic prostate cancer patients was associated with a prolonged overall survival, however the magnitude of previous trial-based survival benefits could not be reassured in real-life population-based data.

## Data Availability Statement

The original contributions presented in the study are included in the article/supplementary material. Further inquiries can be directed to the corresponding author.

## Ethics Statement

Ethical review and approval were not required for the study on human participants in accordance with the local legislation and institutional requirements. The patients/participants provided their written informed consent to participate in this study.

## Author Contributions

BHoe - conceptualization, methodology, formal analysis, writing original draft, writing review and editing, and visualization. FrC – writing review and editing and visualization. CW: writing review and editing and visualization. RF: writing review and editing and visualization. BHor: writing review and editing and visualization. GS: writing review and editing and visualization. ZT: methodology, software, validation, formal analysis, and resources. FS: writing review and editing and supervision. MaG - writing review and editing and supervision. MiG: writing review and editing and supervision. LK: writing review and editing and visualization. PM -writing review and editing and supervision. AB - writing review and editing and supervision. DT - writing review and editing and supervision. FeC: writing review and editing and supervision. SS: writing review and editing and supervision. CT: writing review and editing and supervision. PK: writing review and editing, supervision, project administration, and conceptualization. All authors contributed to the article and approved the submitted version.

## Conflict of Interest

The authors declare that the research was conducted in the absence of any commercial or financial relationships that could be construed as a potential conflict of interest.

## Publisher’s Note

All claims expressed in this article are solely those of the authors and do not necessarily represent those of their affiliated organizations, or those of the publisher, the editors and the reviewers. Any product that may be evaluated in this article, or claim that may be made by its manufacturer, is not guaranteed or endorsed by the publisher.
